# Supplementary material for: Protocerebral Bridge Neurons That Regulate Sleep in Drosophila melanogaster
Source: Front Neurosci. 2021 Oct 15;15:647117. doi: 10.3389/fnins.2021.647117 (PMC8554056; doi:10.3389/fnins.2021.647117)
Supplement: Supplementary file 10 [file Data_Sheet_1.pdf]

Supplementary Material

1 Supplementary Figures

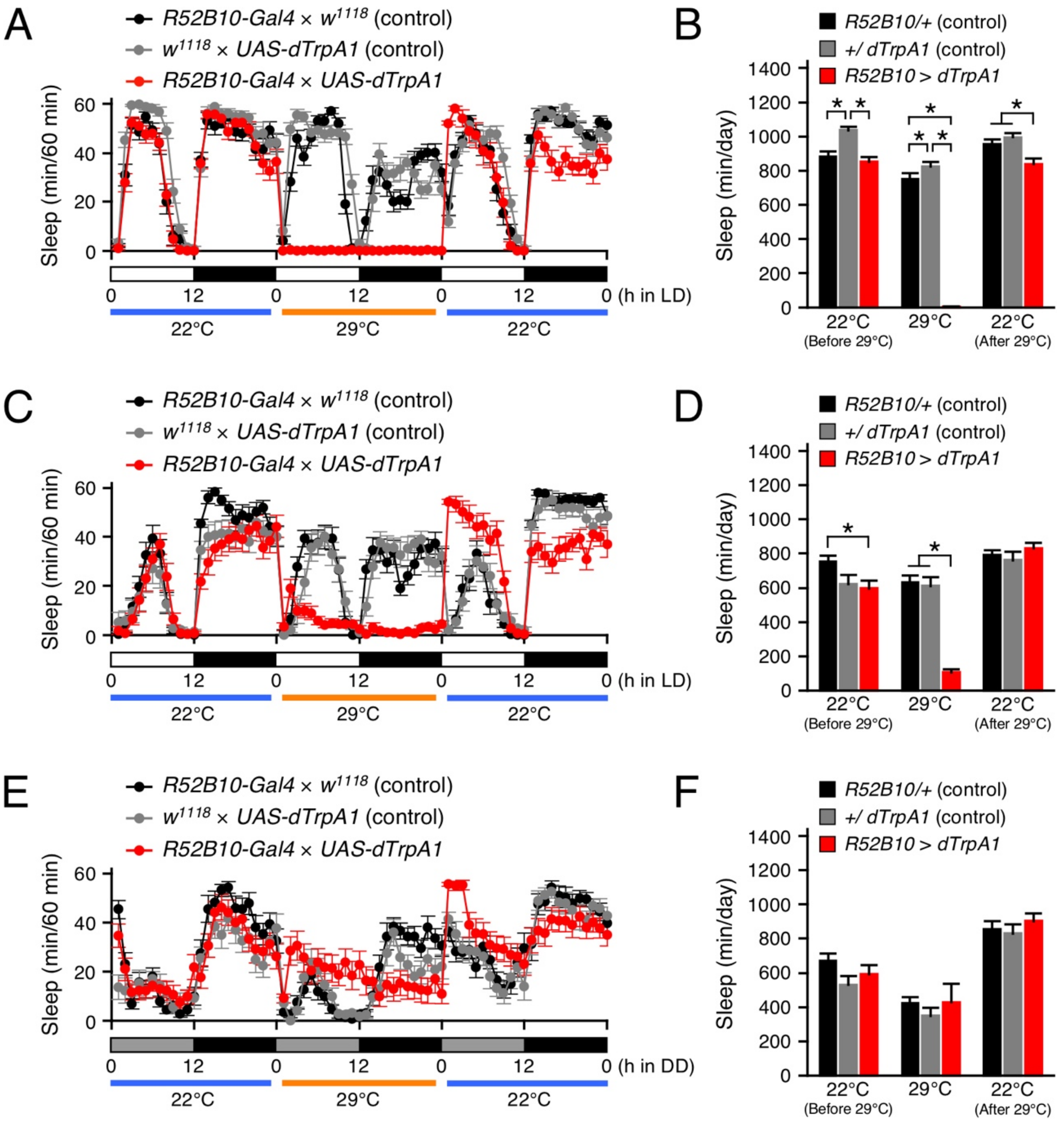

Supplementary Figure 1. Activation of *R52B10-Gal4*-expressing neurons in male and female flies under LD conditions decreased the amount of sleep. **(A,B)** Sleep profiles for 60-min intervals **(A)** or total daily sleep **(B)** for control male flies (*R52B10-Gal4* × w^{1118}, black circles or bars; w^{1118} × *UAS-dTrpA1*, gray circles or bars) or experimental male flies (*R52B10-Gal4* × *UAS-dTrpA1*, red circles or bars) in LD conditions. **(C-F)** Sleep profiles for 60-min intervals **(C,E)** or total daily sleep **(D,F)** for controls or experimental female flies in LD **(C,D)** and DD **(E,F)** conditions. Data are presented as

mean ± SEM (n = 15-16 flies per group). *$p < 0.05$; two-way repeated measures ANOVA followed by Holm multiple comparison test.

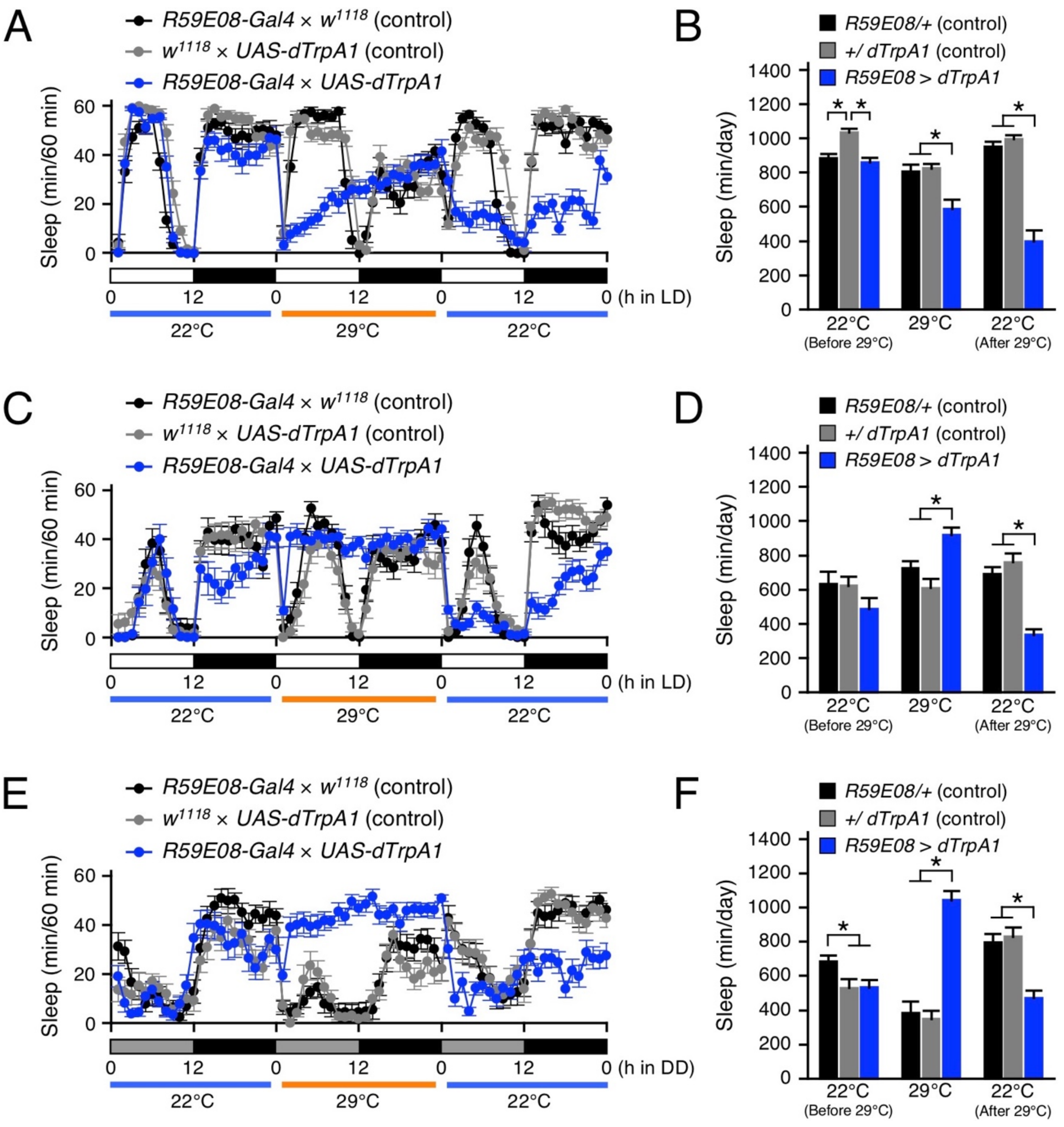

Supplementary Figure 2. Activation of *R59E08-Gal4*-expressing neurons in female flies in LD and DD conditions increased the amount of sleep. **(A,B)** Sleep profiles for 60-min intervals **(A)** or total daily sleep **(B)** for control male flies (*R59E08-Gal4* × w^{1118}, black circles or bars; w^{1118} × *UAS-dTrpA1*, gray circles or bars) or experimental male flies (*R59E08-Gal4* × *UAS-dTrpA1*, blue circles or bars) in LD conditions. **(C-F)** Sleep profiles for 60-min intervals **(C,E)** or total daily sleep (D,F) for controls or experimental female flies in LD **(C,D)** and DD **(E,F)** conditions. Data are presented as mean ± SEM (n = 15-16 flies per group). *$p < 0.05$; two-way repeated measures ANOVA followed by Holm multiple comparison test.

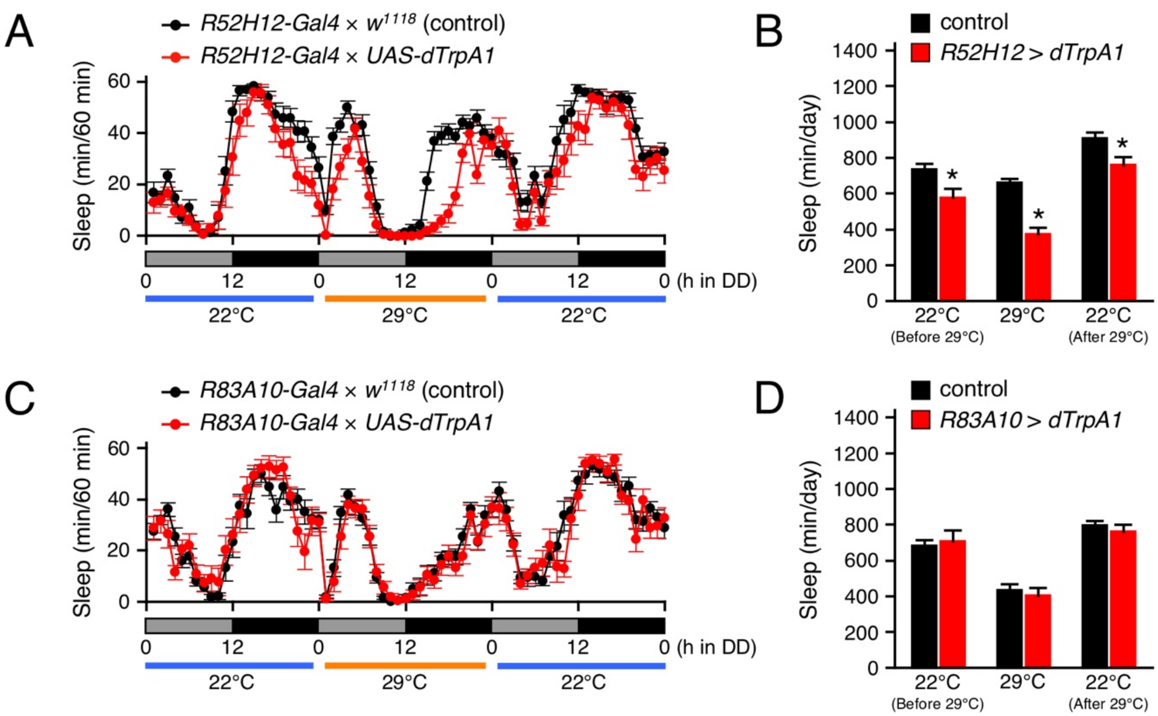

Supplementary Figure 3. Neuronal activation using *Gal4* drivers that express in the AVLP did not remarkably decrease sleep. **(A,B)** Sleep profiles for 60-min intervals **(A)** or total daily sleep **(B)** for controls (*R52H12-Gal4* × w^{1118}, black circles or bars, n = 16) or flies expressing *dTrpA1* with *R52H12-Gal4* (*R52H12-Gal4* × *UAS-dTrpA1*, red circles or bars, n = 14) in DD. The behavior was monitored as described in **Figure 2**. **(C,D)** Sleep profiles for 60-min intervals **(C)** or total daily sleep **(D)** for controls (*R83A10-Gal4* × w^{1118}, black circles or bars, n = 16) or flies expressing *dTrpA1* with *R83A10-Gal4* (*R83A10-Gal4* × *UAS-dTrpA1*, red circles or bars, n = 11) in DD. Data are presented as mean ± SEM. *$p < 0.05$ vs. control at the corresponding temperature conditions (two-way repeated measures ANOVA followed by an analysis of simple main effect).

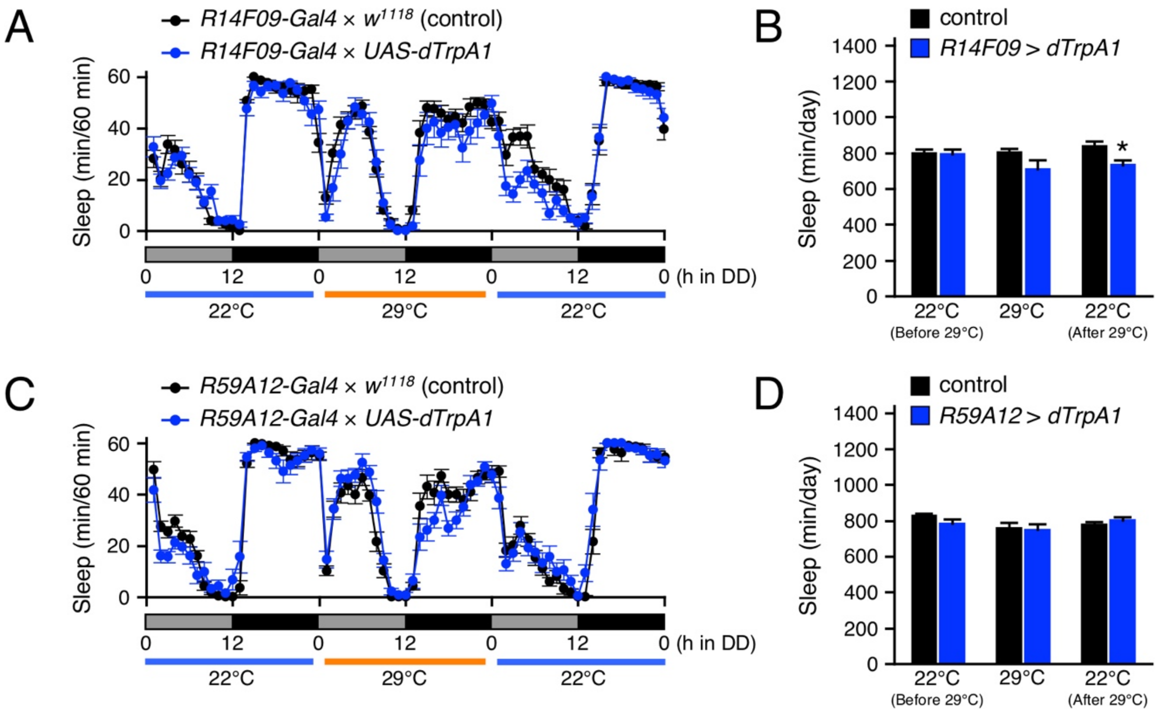

Supplementary Figure 4. Neuronal activation using *Gal4* drivers that express in the lobula plate cells did not increase sleep. **(A,B)** Sleep profiles for 60-min intervals **(A)** or total daily sleep **(B)** for controls (*R14F09-Gal4* × w^{1118}, black circles or bars, *n* = 16) or flies expressing *dTrpA1* with *R14F09-Gal4* (*R14F09-Gal4* × *UAS-dTrpA1*, blue circles or bars, *n* = 16) in DD. The behavior was monitored as described in **Figure 2**. **(C,D)** Sleep profiles for 60-min intervals **(C)** or total daily sleep **(D)** for controls (*R59A12-Gal4* × w^{1118}, black circles or bars, *n* = 15) or flies expressing *dTrpA1* with *R59A12-Gal4* (*R59A12-Gal4* × *UAS-dTrpA1*, blue circles or bars, *n* = 16) in DD. Data are presented as mean ± SEM. *$p < 0.05$ vs. control at the corresponding temperature conditions (two-way repeated measures ANOVA followed by an analysis of simple main effect).

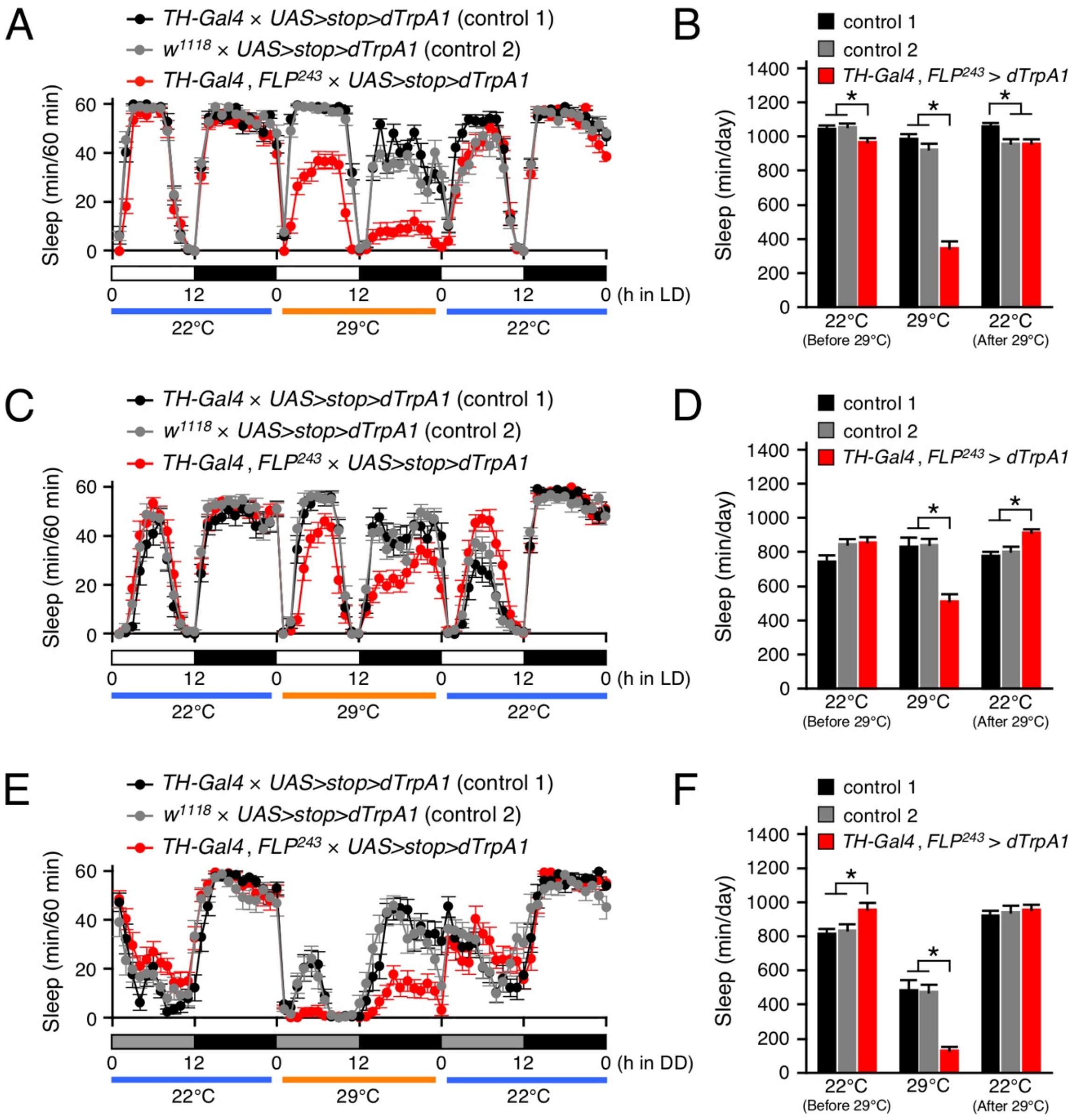

Supplementary Figure 5. Activation of T1 dopaminergic neurons in males under LD and in females under LD and DD decreased the amount of sleep. **(A,B)** Sleep profiles for 60-min intervals **(A)** or total daily sleep **(B)** for control male flies (*TH-Gal4* × *UAS>stop>dTrpA1*, black circles or bars; w^{1118} × *UAS>stop>dTrpA1*, gray circles or bars) or experimental male flies (*TH-Gal4*, FLP^{243} × *UAS>stop>dTrpA1*, red circles or bars) in LD conditions. **(C-F)** Sleep profiles for 60-min intervals **(C,E)** or total daily sleep **(D,F)** for controls or experimental female flies in LD **(C,D)** and DD **(E,F)** conditions. Data are presented as mean ± SEM (n = 16 flies per group). *$p < 0.05$; two-way repeated measures ANOVA followed by Holm multiple comparison test.

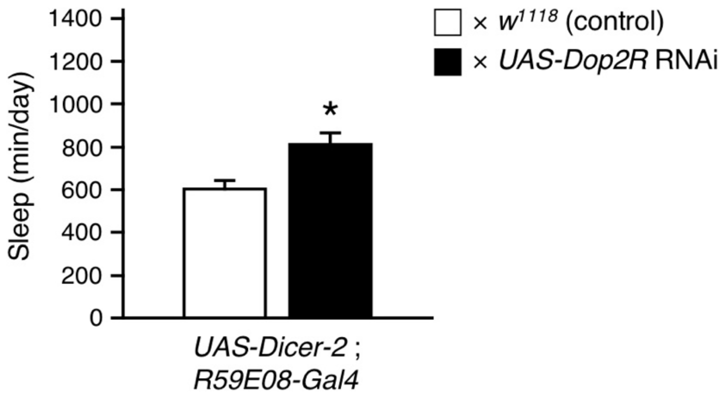

Supplementary Figure 6. *Dop2R* knockdown with *R59E08-Gal4* increased sleep. Total daily sleep for control (*UAS-Dicer-2*; *R59E08-Gal4* × w^{1118}, white bar, n = 13) and *Dop2R* RNAi (VDRC 1820)-expressing flies using the *R59E08-Gal4* driver (*UAS-Dicer-2*; *R59E08-Gal4* × *UAS-Dop2R* RNAi, black bars, n = 13) in DD conditions. Data are averaged across 3 days and are presented as mean ± SEM. *$p < 0.05$; t-test.

2 Supplementary Movies

Supplementary Movie 1. Confocal z-stack of the fly brain expressing *UAS-mCD8::GFP* under the control of *R52B10-Gal4*. Related to Figure 2G left panel.

Supplementary Movie 2. Confocal z-stack of the fly brain expressing *UAS-mCD8::GFP* under the control of *R59E08-Gal4*. Related to Figure 2H.

Supplementary Movie 3. Confocal z-stack of the brains of mosaic flies whose ΔSleep were > +2 SD higher than the mean. Related to #1-7 in Figure 5B.

Supplementary Movie 4. Confocal z-stack of the brains of mosaic flies whose ΔSleep were nearly equal to the mean. Related to #1-7 in Figure 5C.

Supplementary Movie 5. Confocal z-stack of the fly brain expressing *UAS-DenMark* and *UAS-syt-GFP* under the control of *R52B10-Gal4*. Related to Figure 6A.

Supplementary Movie 6. Confocal z-stack of the fly brain expressing *pre-t-GRASP* with *R59E08-Gal4* and *post-t-GRASP* with *R52B10-LexA*. Related to Figure 6B.

Supplementary Movie 7. Confocal z-stack of the brain of *TH-Gal4*, FLP^{243} crossed to *UAS>stop>mCD8::GFP* flies. Related to Figure 7A.

Supplementary Movie 8. Confocal z-stack of the fly brain expressing *CD4-spGFP1-10* with *TH-Gal4* and *CD4-spGFP11* with *R59E08-LexA*. Related to Figure 7D.

Supplementary Movie 9. Confocal z-stack of the fly brain expressing *CD4-spGFP1-10* with *TH-Gal4* and *CD4-spGFP11* with *R52B10-LexA*. Related to Figure 7E.
